# Presidential Symposium: Social Engagement, Isolation, and Loss During the COVID-19 Pandemic

**DOI:** 10.1093/geroni/igab046.643

**Published:** 2021-12-17

**Authors:** Toni Antonucci, Debra Umberson, Deborah Carr

## Abstract

The COVID-19 pandemic has transformed social life across the globe and had a particularly profound impact on older people. In this BSS Presidential Symposium, we address the annual meeting’s theme—Disruption to Transformation: Aging in the “New Normal”—by inviting noted experts to address implications of the COVID-19 pandemic for social engagement and isolation. The speakers address this theme from sociological, psychological, demographic, and public health perspectives with attention to racial disparities and the impact of the pandemic on patterns of isolation and loneliness in the population, methods for conducting research with older populations, patterns of bereavement and loss of family ties, and caregiver mental health. The panel will shed light on these issues through individual presentations and dialogue.

